# Optimization of Squalene Production by *Pseudozyma* sp. P4-22

**DOI:** 10.3390/molecules30071646

**Published:** 2025-04-07

**Authors:** Chen Huang, Xiaojin Song, Jingyi Li, Qiu Cui, Pengfei Gu, Yingang Feng

**Affiliations:** 1 School of Biological Science and Technology, University of Jinan, Jinan 250022, China; 2 CAS Key Laboratory of Biofuels, Shandong Provincial Key Laboratory of Synthetic Biology, Shandong Engineering Laboratory of Single Cell Oil, Qingdao Institute of Bioenergy and Bioprocess Technology, Chinese Academy of Sciences, 189 Songling Road, Qingdao 266101, China; 3 Qingdao Engineering Laboratory of Single Cell Oil, Qingdao New Energy Shandong Laboratory, 189 Songling Road, Qingdao 266101, China; 4 Shandong Energy Institute, 189 Songling Road, Qingdao 266101, China; 5 Haide College, Ocean University of China, Qingdao 266101, China; 6 University of Chinese Academy of Sciences, Beijing 100049, China

**Keywords:** squalene, *Pseudozyma* sp., mutagenesis, culture optimization, fermentation, corn steep liquor

## Abstract

Squalene is an important bioactive substance widely used in the food, pharmaceutical, and cosmetic industries. Microbial production of squalene has gained prominence in recent years due to its sustainability, safety, and environmental friendliness. In this study, a mutant strain, *Pseudozyma* sp. P4-22, with enhanced squalene-producing ability, was obtained through atmospheric and room temperature plasma mutagenesis of the previously screened squalene-producing yeast *Pseudozyma* sp. SD301. The P4-22 strain demonstrated the ability to produce squalene using various carbon and nitrogen sources. We optimized the culture conditions by employing cost-effective corn steep liquor as the nitrogen source, and the optimal pH and sea salt concentration of the medium were determined to be 5.5 and 5 g/L, respectively. Under optimal cultivation conditions, the biomass and squalene production reached 64.42 g/L and 2.06 g/L, respectively, in a 5 L fed-batch fermentation. This study highlights the potential of *Pseudozyma* sp. P4-22 as a promising strain for commercial-scale production of squalene.

## 1. Introduction

Squalene (C_30_H_50_) is a non-saponifiable lipid with six trans double bonds, belonging to the terpenoid family, and serves as a key precursor to fat-soluble vitamins, hormones, and cholesterol [1]. Squalene protects human skin from lipid oxidation caused by ultraviolet radiation or other oxidative stress [2], significantly reducing free radical transmission on the skin and thereby shielding cellular DNA from damage [3]. Like polyunsaturated fatty acids, squalene is a key component of the skin and is widely utilized in skincare products due to its moisturizing and antioxidant properties [4]. Squalene plays a significant role in generating antigen-specific T cells by transporting antigens alongside neutrophils and IL-18, thereby contributing to immune system modulation [5]. Owing to its stability and biocompatibility, squalene is employed in vaccine and drug delivery emulsions. For example, adjuvants like MF59 and AS03, which contain squalene, have been used in human influenza vaccines [6]. Following the outbreak of COVID-19 in 2019, squalene has been employed as an adjuvant in vaccines designed to combat the novel coronavirus [7,8]. As such, squalene possesses numerous beneficial properties including antioxidative [2,4], anticancer [9,10,11], anti-aging [12,13], and anti-inflammatory [9,14,15], as well as being used in the treatment of skin diseases [16] and cardiovascular health maintenance [9]. It is widely used across various sectors, such as food, pharmaceuticals, and cosmetics [17,18,19]. Market reports indicate that the global market size for squalene was valued at 129 million US dollars in 2020 and is expected to grow at a compound annual growth rate of 7.3%, reaching 184 million US dollars by 2025 [4].

The primary methods for squalene production include animal/plant extraction and microbial fermentation [4]. However, growing concerns regarding ecological impacts, resource limitations, and sustainability challenges associated with traditional extraction methods have shifted the research focus toward microbial fermentation as a more viable production strategy [17,20]. Microbial production of squalene utilizes two distinct metabolic pathways [21]. In prokaryotes like *Escherichia coli*, squalene is typically synthesized via the 2-methyl-D-erythritol 4-phosphate (MEP) pathway. In eukaryotes, such as yeast, fungi, microalgae, and animals, squalene is synthesized using the mevalonate acid (MVA) pathway. The MVA pathway utilizes acetyl-CoA as a precursor, and key enzymes such as 3-hydroxy-3-methylglutaryl-CoA synthase (HMGS), 3-hydroxy-3-methylglutaryl-CoA reductase (HMGR), mevalonate kinase, mevalonate-5-phosphate kinase, and diphosphomevalonate decarboxylase are employed to synthesize isopentenyl pyrophosphate (IPP). Both the MEP and MVA pathways ultimately isomerize IPP into dimethylallyl pyrophosphate (DMAPP) through an IPP isomerase. IPP and DMAPP then condense to form farnesyl pyrophosphate (FPP). Finally, two molecules of FPP are utilized to synthesize squalene through squalene synthase (SQS) [4].

While wild-type squalene-producing strains exhibit inherent environmental adaptability and biosafety advantages, their industrial application remains constrained by limitations including low yield, process instability, and long production cycles. Natural producers of squalene mainly include yeasts, *Aurantiochytrium* spp., and certain microalgae (see examples in Table 1). Among these, *Pseudozyma* spp. are yeasts of notable commercial potential, known for their ability to produce glycolipid biosurfactants, squalene, isocitric acid, and extracellular polysaccharides [22]. In our previous study, we identified the squalene-producing *Pseudozyma* sp. strain SD301 [23]. Subsequent research successfully performed electroporation on mutant strains derived from SD301 [24], demonstrating that genetic modification of *Pseudozyma* sp. strains is also feasible.

**Table 1:** Some wild-type strains producing squalene reported in the literature.

Strains	Squalene Titer	Media or Culture Method	Reference
*Rhodosporidium* sp. DR37	619 mg/L	modified YEPD media	[25]
*Saccharomyces cerevisiae* EGY48	3129 μg/L	glucose, soy peptone, and yeast extract, under semi-aerobic conditions	[26]
*Torulaspora delbrueckii*	237.25 μg/gDW	molasses	[27]
*Pseudozyma* sp. JCC207	340.52 mg/L	glucose, yeast extract, sea salt	[28]
*Saccharomyces cerevisiae* EGY48	20.70 mg/L	glucose, soy peptone, and yeast extract with 0.442 mM terbinafine and 0.044 mM methyl jasmonate	[29]
*Schizochytrium* sp. HX-308	439.98 mg/L	glucose and yeast extract; agitation and ventilation rates were set at 250 rpm and 0.6 m^3^h^−1^	[30]
*Thraustochytrium* striatum N5997	13 mg/gDW	glucose and yeast extract	[31]
*Thraustochytrium* ATCC 26185	72.9 mg/L	glucose and yeast extract with mannitol	[32]
*Aurantiochytrium* sp. TWZ-97	240.2 mg/L	glucose, yeast extract, 0.7 g/L α-tocopherol	[33]
*Aurantiochytrium* sp. Yonez 5−1	1073.66 mg/L	glucose, tryptone, yeast extract	[34]
*Aurantiochytrium* sp. 18W-13a	1.29 g/L	glucose, proteose-peptone, yeast extract	[35]
*Thraustochytrium* MST1253	65.2 mg/L	glucose, peptone, yeast extract	[36]
*Thraustochytrium* sp. MAN37 FRU	10.2 mg/gDW	peptone, yeast extract, glucose	[37]
*Cutaneotrichosporon oleaginosus*	367.89 mg/L	glucose, urea, peptone, yeast extract, 10 mg/L terbinafine	[38]
*Phormidium autumnale*	0.18 g/kgDW	glucose and slaughterhouse wastewater	[39]
*Pseudozyma* sp. SD 301	2.45 g/L	glucose and yeast extract	[23]
*Pseudozyma* sp. P4-22	2.06 g/L	glucose and corn steep liquor	This study

To enhance the economic viability of fermentative squalene production, this study employed atmospheric and room temperature plasma (ARTP) mutagenesis to generate and screen mutants of *Pseudozyma* sp. SD301 and successfully isolated a mutant strain, *Pseudozyma* sp. P4-22, with enhanced squalene production capacity. Subsequently, optimization of media and culture conditions was conducted using cost-effective corn steep liquor as a nitrogen source.

## 2. Results and Discussion

### 2.1. ARTP Mutagenesis

**Figure 1:**
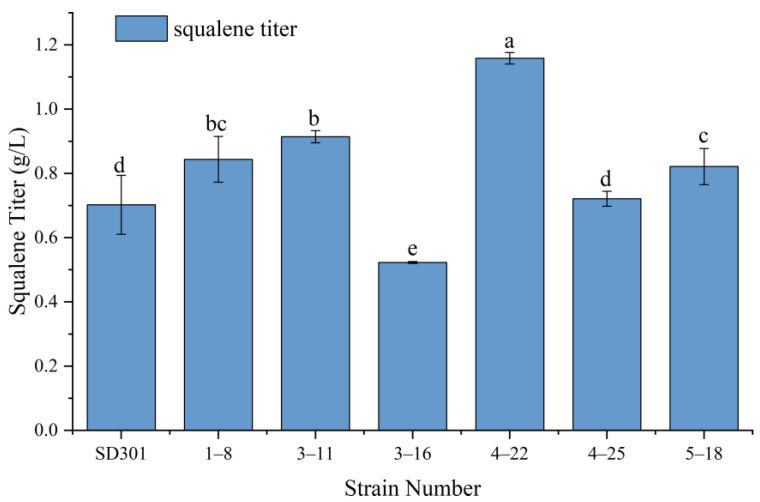
Squalene titer of selected strains obtained by ARTP mutagenesis. Different lowercase letters (a, b, c, d, e) above bars indicate statistically significant differences between means (*p* < 0.05), while shared letters denote no significant difference.

**Figure 2:**
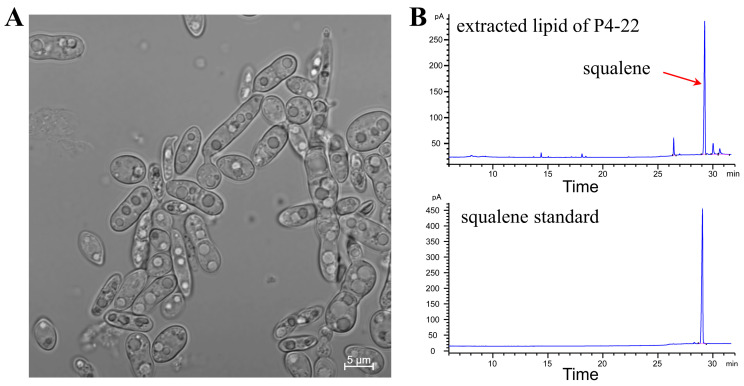
The optical image of *Pseudozyma* sp. P4-22 (**A**) and the gas chromatography spectrum of extracted lipid (**B**).

ARTP mutagenesis is a plasma-based technique for inducing genetic mutations in microorganisms, plants, or other biological organisms [40]. Compared with traditional methods, this powerful mutagenesis approach offers several advantages including environmental friendliness, mild operation conditions, and rapid mutation rates. Consequently, ARTP mutagenesis, particularly when combined with high-throughput screening technologies, has found widespread application in microbiology [41,42]. In this study, we employed ARTP technology to generate mutant strains of *Pseudozyma* sp. SD301. From a total of 97 isolated colonies, we identified six strains exhibiting elevated squalene titers through shake-flask experiments (Figure 1). Among these, the highest-producing strain was selected and designated as P4-22. Figure 2 presents both the morphological appearance of P4-22 colonies and the characteristic gas chromatography profile of its extracted lipids. Notably, to the best of our knowledge, *Pseudozyma* sp. SD301 previously represented the highest reported titer among non-genetically modified microorganisms (Table 1). The superior performance of P4-22 therefore warrants further investigation.

### 2.2. Effect of Carbon Source on P4-22 Fermentation Performance

To investigate the influence of carbon sources on the growth and squalene accumulation of strain P4-22, we evaluated fifteen carbon sources in cultivation experiments. As shown in Table 2, each carbon source exhibited distinct effects on both cell growth and squalene production. Glucose emerged as the optimal carbon source, supporting the highest biomass (21.50 ± 0.18 g/L) and squalene titer (0.99 ± 0.03 g/L). Xylose ranked second, yielding lower biomass (17.79 ± 0.75 g/L) but comparable squalene production (0.98 ± 0.03 g/L), suggesting P4-22’s potential for utilizing cost-effective substrates like lignocellulose hydrolysate [43,44]. When sucrose was used as the carbon source, the biomass reached 19.98 ± 0.32 g/L, with a squalene titer of 0.82 ± 0.07 g/L. Other carbon sources, including maltose, lactose, succinic acid, and ethanol, enabled modest squalene accumulation, although significantly lower than that of glucose or xylose. The remaining tested carbon sources proved ineffective for squalene production.

Previous studies have demonstrated that most *Pseudozyma* species possess xylose metabolic capability, although utilization efficiency varies substantially among strains. For biosurfactant production, Faria et al. reported that *Pseudozyma antarctica* PYCC 5048^T^ produced mannosylerythritol lipids (MEL) at similar titers from xylose and glucose, while *Pseudozyma aphidis* PYCC 5535^T^ showed markedly lower production on xylose [45]. Meirke et al. observed that the oleaginous yeast *Pseudozyma hubeiensis* BOT-O consumed glucose and xylose at comparable rates in single-substrate media, but exhibited preferential glucose utilization in mixed sugar conditions [46]. These findings highlight the need for further investigation into xylose metabolism mechanisms in strain P4-22.

### 2.3. Effect of Nitrogen Source on P4-22 Fermentation Performance

Nitrogen sources play a significant role in the growth and fermentation activity of microorganisms. In this study, eight nitrogen sources were tested for their effects on the growth and squalene production of the P4-22 strain (Table 3). The results showed that P4-22 performed better with organic nitrogen sources than with inorganic ones. Among them, corn steep liquor was the most effective, giving the highest biomass (23.28 ± 0.45 g/L) and squalene titer (1.74 ± 0.003 g/L). Yeast extract gave slightly lower results, with biomass at 23.07 ± 1.44 g/L and squalene titer at 1.29 ± 0.11 g/L. Tryptone supported only small amounts of squalene production. No squalene accumulation was observed with any inorganic nitrogen sources, and cell growth was also poor under these conditions. Therefore, corn steep liquor was selected as the best nitrogen source for P4-22 fermentation. Moreover, since corn steep liquor is a low-cost nitrogen source suitable for industrial production, it was chosen for further optimization of fermentation conditions.

Previous studies on squalene production mainly used yeast extract or peptone as nitrogen sources (Table 1). However, for industrial fermentation, cheaper nitrogen sources such as inorganic salts or corn steep liquor are preferred to reduce costs. Because P4-22 cannot use inorganic nitrogen sources efficiently, corn steep liquor is the best choice for further investigation.

**Figure 3:**
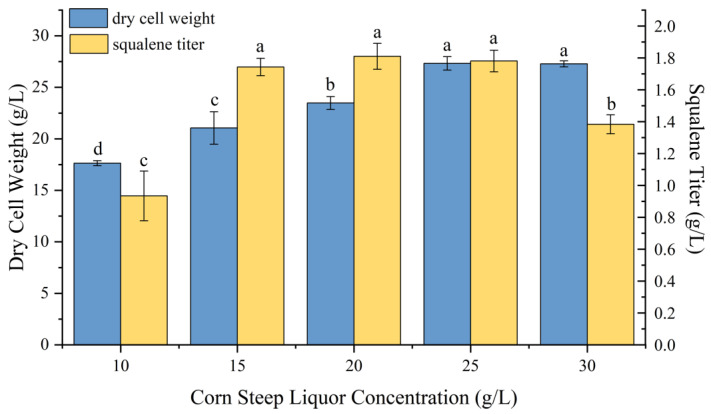
Effects of different corn steep liquor concentrations on growth and squalene production of P4-22. Different lowercase letters (a, b, c, d) above bars indicate statistically significant differences between means (*p* < 0.05), while shared letters denote no significant difference.

To determine the optimal nitrogen source concentration for strain P4-22, we tested various concentrations of corn steep liquor (10 g/L, 15 g/L, 20 g/L, 25 g/L, and 30 g/L), corresponding to carbon-to-nitrogen ratios of 6, 4, 3, 2.4, and 2, respectively. The results are presented in Figure 3. Biomass increased with higher nitrogen concentrations, reaching a maximum of 27.33 ± 0.65 g/L at 25 g/L of corn-steep liquor. Meanwhile, squalene production peaked at a corn-steep liquor concentration of 20 g/L, achieving a titer of 1.8 ± 0.08 g/L.

### 2.4. Effect of pH on P4-22 Fermentation

**Figure 4:**
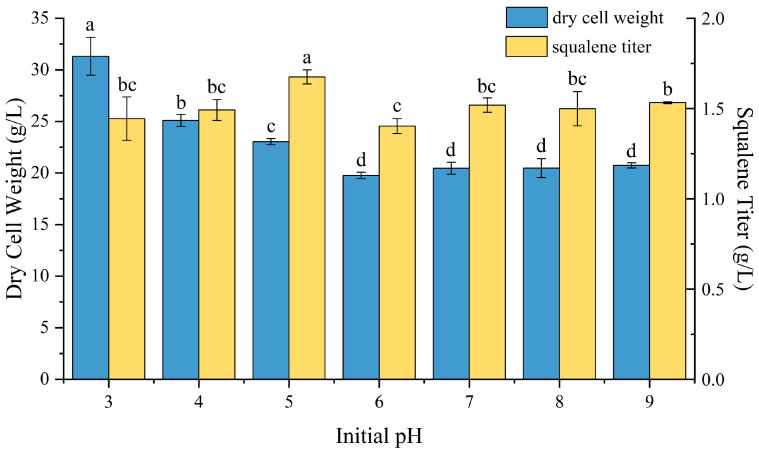
Effects of different initial pH on strain growth and accumulation of squalene. Different lowercase letters (a, b, c, d) above bars indicate statistically significant differences between means (*p* < 0.05), while shared letters denote no significant difference.

The initial pH of the culture medium is a crucial parameter in microbial fermentation, significantly affecting cell growth, metabolic activity, and product formation [47,48,49]. We examined the impact of initial pH (ranging from 3 to 9) on P4-22 growth and squalene production (Figure 4). The strain demonstrated broad pH adaptability, growing across the entire tested range. Notably, biomass accumulation showed an inverse relationship with pH, reaching maximum levels (31.31 ± 1.83 g/L) at pH 3 and gradually decreasing to 20.74 ± 0.25 g/L at pH 9. Squalene production peaked at pH 5 (1.68 ± 0.04 g/L), with corresponding biomass of 23.04 ± 0.3 g/L. pH monitoring during fermentation revealed that all cultures, except those with initial pH 3–4, reached approximately pH 5.3 by day 2. By the end of fermentation, all cultures stabilized at a final pH of around 5.5. These results indicate that the optimum pH for P4-22 fermentation is approximately 5.5.

### 2.5. Effect of Sea Salt Concentration on P4-22 Fermentation

**Figure 5:**
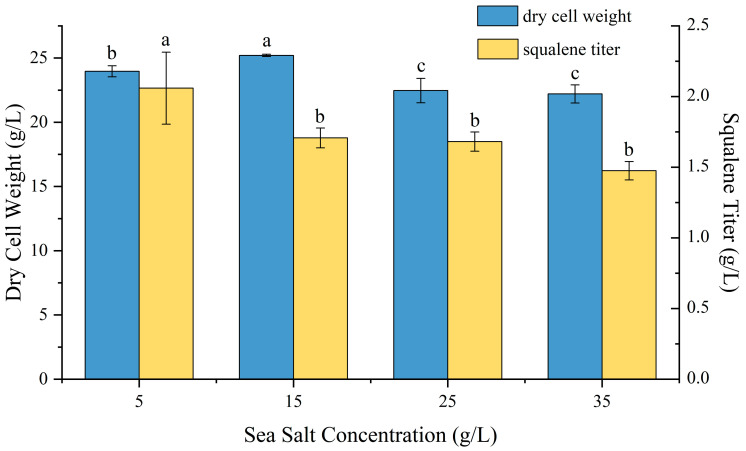
The growth and squalene accumulation of P4-22 at different salt concentrations. Different lowercase letters (a, b, c) above bars indicate statistically significant differences between means (*p* < 0.05), while shared letters denote no significant difference.

Salt concentration plays a significant role in microbial growth, metabolism, and product formation [50,51,52]. To examine the effects of sea salt concentrations on P4-22 growth and squalene production, we conducted fermentations with sea salt concentrations ranging from 5 to 35 g/L (Figure 5). The results demonstrated that P4-22 could grow and produce squalene across all tested concentrations, although squalene accumulation was more favorable under lower-salt conditions. Maximum biomass (24.52 ± 0.22 g/L) occurred at 15 g/L of sea salt, with a corresponding squalene titer of 1.66 ± 0.05 g/L. Notably, the highest squalene production (2 g/L) was achieved at the lowest tested concentration 5 g/L. Based on these results, the optimal sea salt concentration for squalene production by P4-22 was determined to be 5 g/L. This represents a significant difference from the original SD301 strain, which showed optimal performance at 15 g/L of sea salt [23].

### 2.6. Fed-Batch Fermentation Experiments for Squalene Production

**Figure 6:**
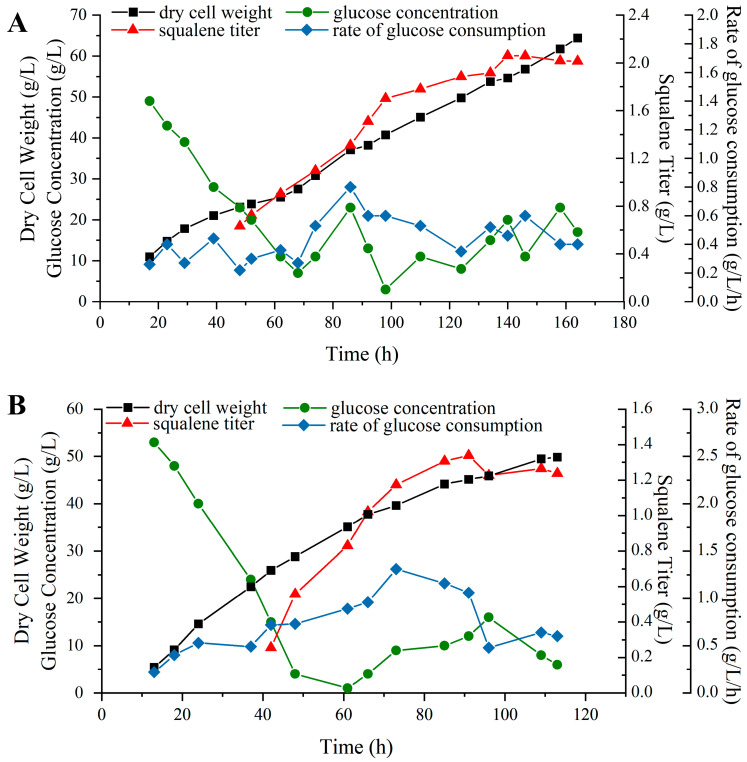
Fed-batch fermentations of *Pseudozyma* sp. P4-22 (**A**) and *Pseudozyma* sp. SD301 (**B**).

Scaling-up culture represents a critical step in transitioning laboratory-scale microbial fermentation processes to industrial-scale production. Based on our optimization results, we performed fed-batch fermentation using strain P4-22 (Figure 6A). Glucose supplementation started at 68 h, with a total of 310 mL glucose consumed during 164 h of fermentation. The process yielded a maximum biomass of 64.42 g/L and a squalene titer of 2.06 g/L. As a control, strain SD301 was fermented in a 5 L fermenter using corn steep liquor as a nitrogen source (Figure 6B). After 113 h, this strain achieved 49.83 g/L biomass and 1.34 g/L squalene titer. Under optimized conditions, P4-22 demonstrated approximately 1.5-fold higher squalene production compared to the original SD301 strain.

Notably, when using cost-effective corn steep liquor, P4-22 achieved production levels comparable to SD301 cultures employing more expensive yeast extract (Table 1) [23]. However, the improved strain required longer fermentation times. Future studies should focus on systematic medium optimization and precise fermentation controls to enhance efficiency. Our research also indicates that fermentation processes employing different low-cost carbon and nitrogen sources should be carefully optimized and studied for different chassis cells, as different strains may exhibit varying adaptability.

## 3. Materials and Methods

### 3.1. Strain and Culture

*Pseudozyma* sp. P4-22 was derived from *Pseudozyma* sp. SD301 (CGMCC No. 9687, China General Microbiological Culture Collection Center) through ARTP mutagenesis and stored in a laboratory freezer at −80 °C. The optical image of P4-22 was acquired using an Olympus FluoView^TM^ FV1000 confocal microscope.

The culture media used in this study include GYS medium (60 g/L glucose [Fufeng Group, Qingdao, China], 20 g/L yeast extract [Angel Yeast Co., Ltd., Yichang, China], 15 g/L sea salt [QianJiang Sea Salt Factory, Weifang, China]) and GCS medium (60 g/L glucose, 20 g/L corn steep liquor [Angel Yeast Co., Ltd., Yichang, China], 5 g/L sea salt). The corn steep liquor also provides the phosphate source since it contains 1% (*w*/*w*) soluble phosphate. For shake flask experiments, a single P4-22 colony was inoculated into 50 mL of GYS medium and cultured at 25 °C for 48 h. Subsequently, a 2% inoculum was transferred to a GCS medium for 6-day fermentation. All cultures were maintained at 200 rpm agitation speed.

### 3.2. ARTP Mutagenesis

Single colonies were inoculated into 30 mL of GYS medium and cultured at 25 °C with 200 rpm shaking for 24 h. A 10 μL aliquot of the culture was uniformly spread onto a sterilized slide, which was then placed in a sterilized Petri dish and transferred to the ARTP chamber. The distance between the slide and the plasma generator jet outlet was maintained at approximately 2–3 mm. The ARTP mutagenesis parameters were set as follows: 120 W working power, 10 L/min air flow rate, 20 °C circulating water temperature, and 90 s treatment duration. After treatment, cells were collected in an EP tube containing 1 mL sterile water, vortexed for 1 min, and serially diluted. A 100 μL aliquot of each dilution was then spread on a GYS agar plate.

### 3.3. Optimization of Cultivation Conditions

To investigate the effects of various chemical and physical parameters on cell growth and squalene accumulation in *Pseudozyma* sp. P4-22, a series of single-factor experiments was conducted. Fifteen carbon sources were tested: glucose, sucrose, maltose, xylose, raffinose, lactose, soluble starch, citric acid, malic acid, succinic acid, glycerol, methanol, ethanol, lactic acid, and sorbitol. Eight nitrogen sources were evaluated: yeast extract, peptone, corn steep liquor, urea, ammonium chloride, ammonium sulfate, sodium nitrate, and potassium nitrate. The effects of varying pH levels (3, 4, 5, 6, 7, 8, 9) and sea salt concentrations (5 g/L, 15 g/L, 25 g/L, 35 g/L) on cell growth and squalene accumulation were investigated.

### 3.4. Fed-Batch Fermentations in a 5 L Fermenter

For scale-up fermentation in a 5 L fermenter, both primary and secondary seed cultures were prepared using a GYS medium. The primary seed culture was prepared by inoculating *Pseudozyma* sp. P4-22 into 50 mL of GYS medium and incubating at 25 °C with 200 rpm agitation for 24 h. The entire primary seed culture was then transferred into 200 mL of fresh GYS medium and cultivated for 48 h at 25 °C with 200 rpm agitation until the OD_600_ reached approximately 8, yielding the secondary seed culture. Fed-batch fermentation was carried out in a 5 L fermenter containing 3.3 L of fermentation medium inoculated with the secondary seed culture. The fermentation conditions were controlled as follows: pH was maintained at 5.5 using 2 M NaOH or 2 M HCl, the temperature was kept constant at 25 °C, and the airflow rate was maintained at 1.4–2.0 vvm. A 40% glucose solution was fed into the fermenter when the initial glucose was depleted, and the glucose concentration in the medium was then maintained below 20 g/L by adjusting the feed rate according to the glucose consumption rate in the previous period.

### 3.5. Analysis of Biomass

For biomass determination, 50 mL aliquots of culture were collected and centrifuged at 5000× *g* for 5 min. The resulting cell pellets were lyophilized for 8 h, and the biomass was quantified by dry weight measurement.

### 3.6. Extraction and Detection of Lipids and Squalene

For lipid extraction, 50 mg of freeze-dried cell powder (M0) was mixed with 600 μL of 6 M HCl in a 2 mL centrifuge tube. After vortexing for 30 s, the mixture was incubated at room temperature for 30 min. Cell disruption was achieved through repeated cycles of alternating boiling (5 min) and freezing (−80 °C, 5 min). Subsequently, 900 μL of chloroform-methanol (2:1, *v*/*v*) was added, followed by vortexing (30 s) and centrifugation (12,000× *g*, 5 min). The lower organic phase was transferred to a new centrifuge tube and mixed with an equal volume of saturated NaCl solution. After vortexing (30 s) and centrifugation (at 12,000× *g*, 5 min) to remove proteins, the lower organic layer was transferred to a pre-weighed glass vial (M1) and dried under vacuum at 55 °C for 30 min. The total lipid content was calculated as (M2 − M1)/M0 × 100%, where M2 represents the vial weight after drying.

The extracted lipids were analyzed for squalene content using gas chromatography. For sample preparation, 1 mL of saturated NaCl solution and 2 mL of n-hexane were added to the glass vial containing the extracted lipids. Following thorough mixing, the upper hexane layer was transferred to a chromatography vial. Analysis was performed on an Agilent Technologies 7890B gas chromatograph equipped with an HP-INNOWAX capillary column (30 m × 0.25 mm × 0.25 μm). The temperature program consisted of an initial hold at 100 °C for 1 min, followed by a temperature ramp at 15 °C/min to 250 °C, with a final hold at 250 °C for 5 min. The injector and detector temperatures were maintained at 280 °C. High-purity nitrogen served as the carrier gas with a split ratio of 1:19, and samples were injected at a volume of 1 μL.

Squalene quantification was achieved by comparing the sample peak area to a standard calibration curve. A series of squalene standard solutions were prepared at dilution factors ranging from 400 to 5000 (400, 500, 800, 1600, 2000, 4000, and 5000). The calibration curve was generated by plotting the peak areas obtained from gas chromatography analysis against the known squalene concentrations of each standard.

### 3.7. Statistical Analysis

All shake-flask experiments were conducted in triplicate, with results expressed as mean values ± standard deviation. The statistical significance of the experimental data was assessed using one-way ANOVA with a significance threshold of *p* < 0.05 for mean comparisons.

## 4. Conclusions

*Pseudozyma* sp. P4-22 exhibited outstanding potential for squalene production under optimized fermentation conditions (25 °C, pH 5.5, with 60 g/L glucose, 20 g/L corn steep liquor, and 5 g/L sea salt). In a 5 L fed-batch fermentation system, the strain achieved a maximum squalene titer of 2.06 g/L, demonstrating robust performance under scale-up conditions. The successful substitution of yeast extract with cost-effective corn steep liquor as the nitrogen source significantly enhances the economic viability of the process. Notably, the ability of P4-22 to utilize xylose for squalene production adds another layer of versatility to this strain, making it adaptable to different carbon sources and potentially expanding its industrial applications. These findings establish P4-22 as a promising microbial platform for squalene production and warrant further investigation into strain optimization and process development to enhance production efficiency and expand potential industrial applications.

## Figures and Tables

**Table 2:** Utilization of different carbon sources by P4-22.

Carbon Source	Strain Biomass (g/L)	Squalene Titer (g/L) ^1^
glucose	21.51 ± 0.18	0.99 ± 0.03
sucrose	19.98 ± 0.32	0.82 ± 0.07
maltose	6.05 ± 0.52	0.05 ± 0.01
xylose	17.79 ± 0.75	0.98 ± 0.03
cellobiose	3.12 ± 0.27	-
lactose	10.27 ± 1.67	0.25 ± 0.17
starch soluble	5.84 ± 0.22	-
citric acid	5.36 ± 0.05	-
malic acid	3.06 ± 1.79	-
succinic acid	12.83 ± 3.49	0.11 ± 0.003
glycerin	5.77 ± 0.08	-
methanol	1.02 ± 0.06	-
ethanol	4.37 ± 0.11	0.003 ± 0.001
lactic acid	3.75 ± 0.03	-
sorbitol	0.96 ± 0.11	-

^1^ “-” represents the absence of squalene accumulation.

**Table 3:** Utilization of different nitrogen sources by P4-22.

Nitrogen Source	Strain Biomass (g/L)	Squalene Titer (g/L) ^1^
yeast extract	23.07 ± 1.44	1.29 ± 0.11
tryptone	12.14 ± 0.16	0.04 ± 0.02
corn steep liquor	23.28 ± 0.45	1.74 ± 0.003
urea	1.03 ± 0.25	-
NH_4_Cl	1.26 ± 0.14	-
(NH_4_)_2_SO_4_	1.54 ± 0.19	-
NaNO_3_	2.1 ± 0.45	-
KNO_3_	1.69 ± 0.09	-

^1^ “-” represents the absence of squalene accumulation.

## Data Availability

Data are contained within the article.

## Abbreviations

The following abbreviations are used in this manuscript:

ARTP	atmospheric and room temperature plasma
MEP	2-methyl-D-erythritol 4-phosphate
MVA	mevalonate acid
HMGS	3-hydroxy-3-methylglutaryl-CoA synthase
HMGR	3-hydroxy-3-methylglutaryl-CoA reductase
IPP	isopentenyl pyrophosphate
DMAPP	dimethylallyl pyrophosphate
FPP	farnesyl pyrophosphate
SQS	squalene synthase
